# Supplementary material for: Variation in the Composition and Quality of Nigella sativa L. Seed Oils—The Underestimated Impact on Possible Health-Promoting Properties
Source: Molecules. 2024 Mar 19;29(6):1360. doi: 10.3390/molecules29061360 (PMC10975171; doi:10.3390/molecules29061360)

Figure S1. Gas chromatographic separation of fatty acids

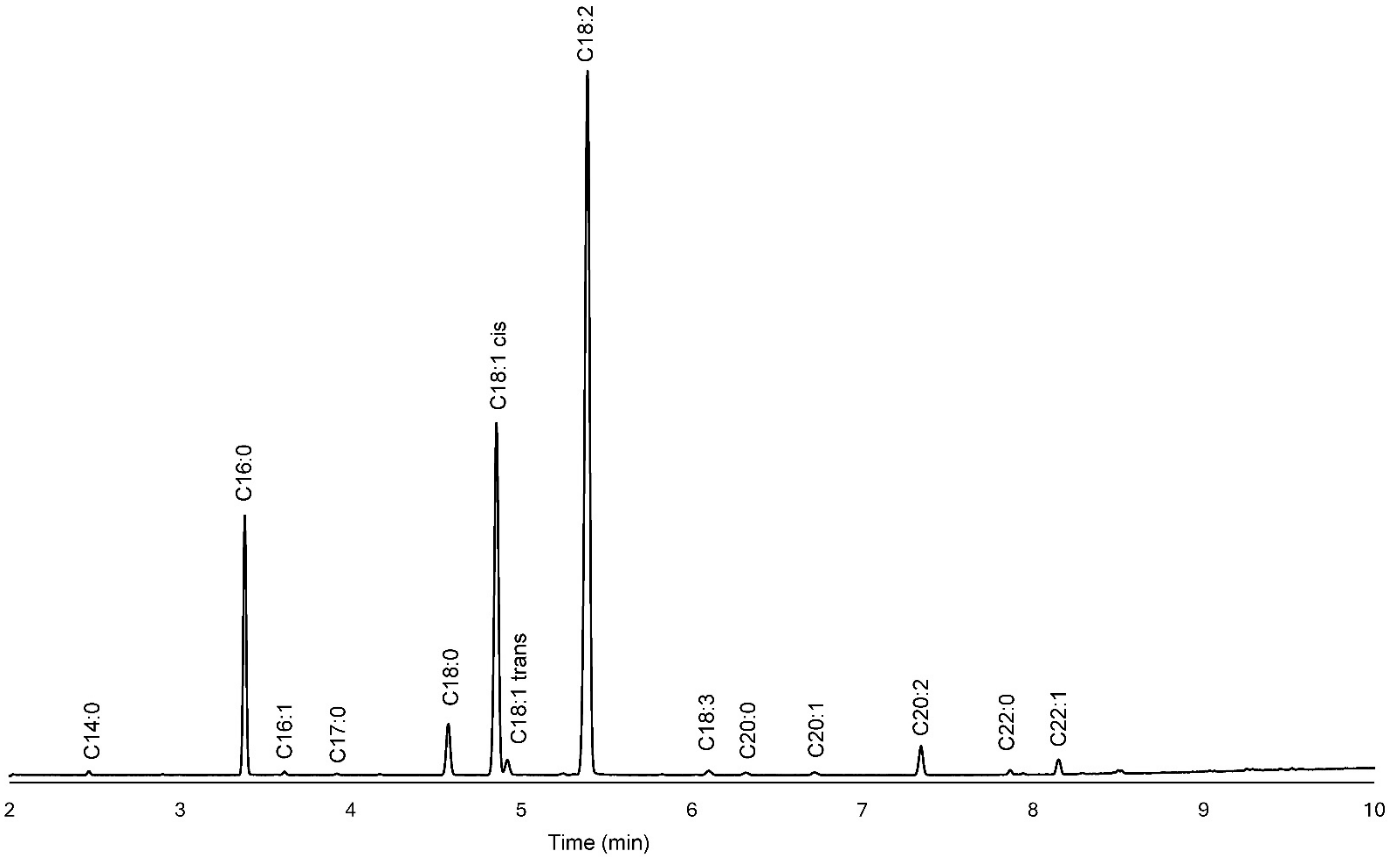

Figure S2. Gas chromatographic separation of squalene and phytosterols

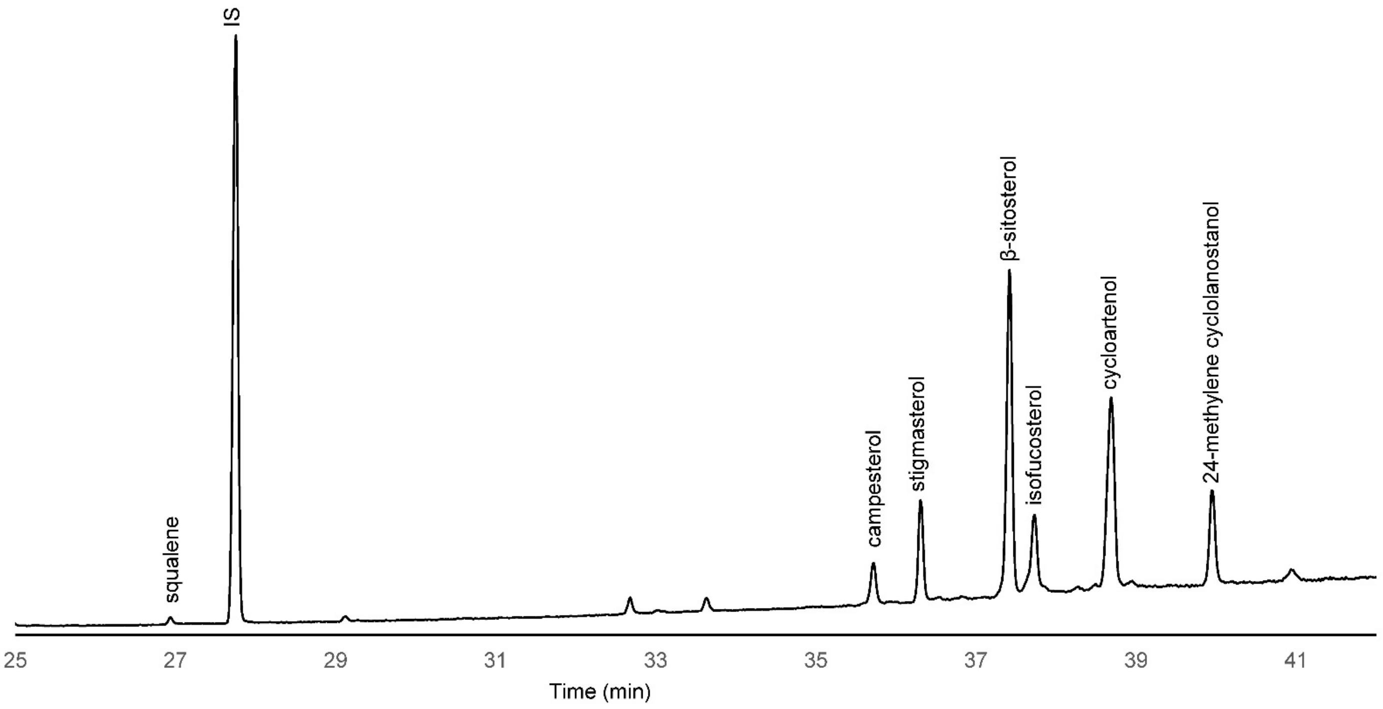

Figure S3. High performance liquid chromatographic separation of tocols

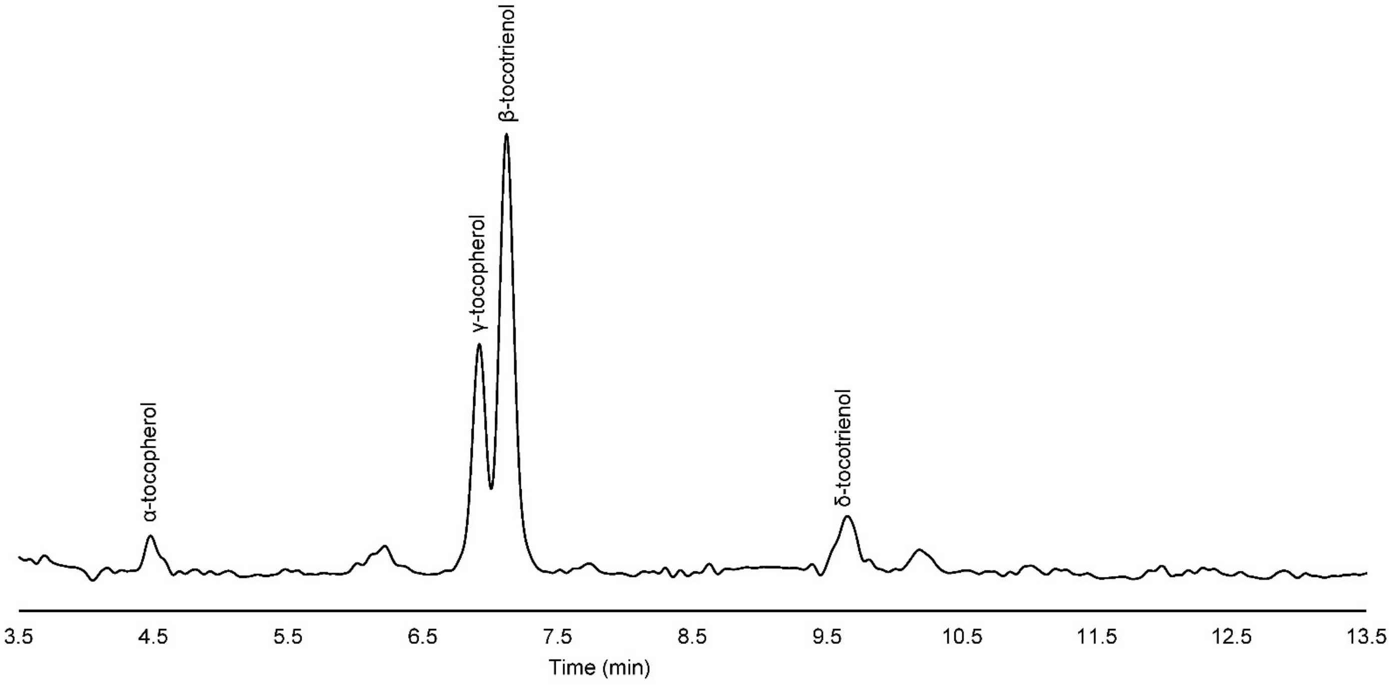

Figure S4. Gas chromatographic separation of volatile compounds

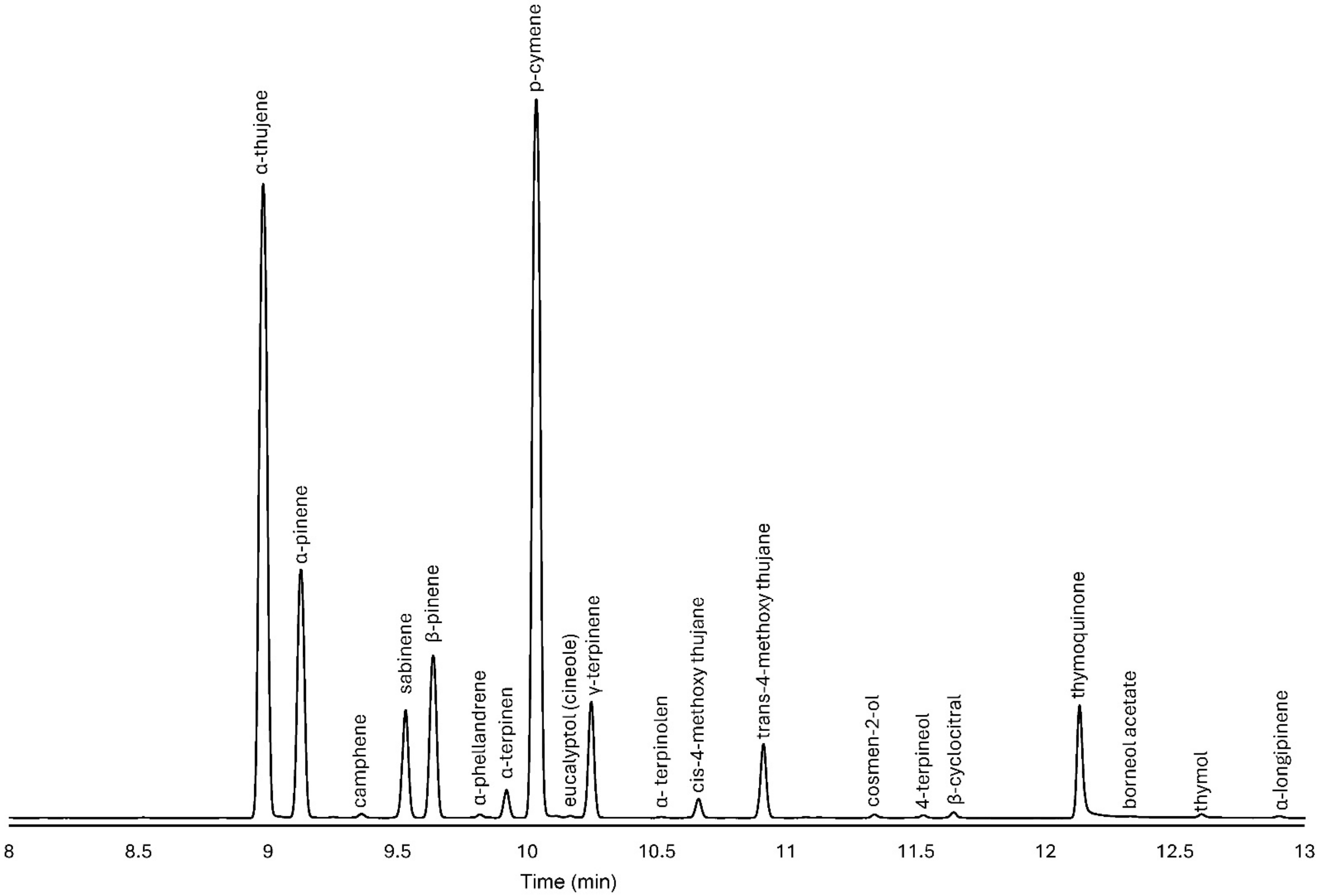

Figure S5. Results of headspace GC-MS analysis of volatile compounds of *N. sativa* L. seeds oils (values are expressed as peak area arbitrary units share).

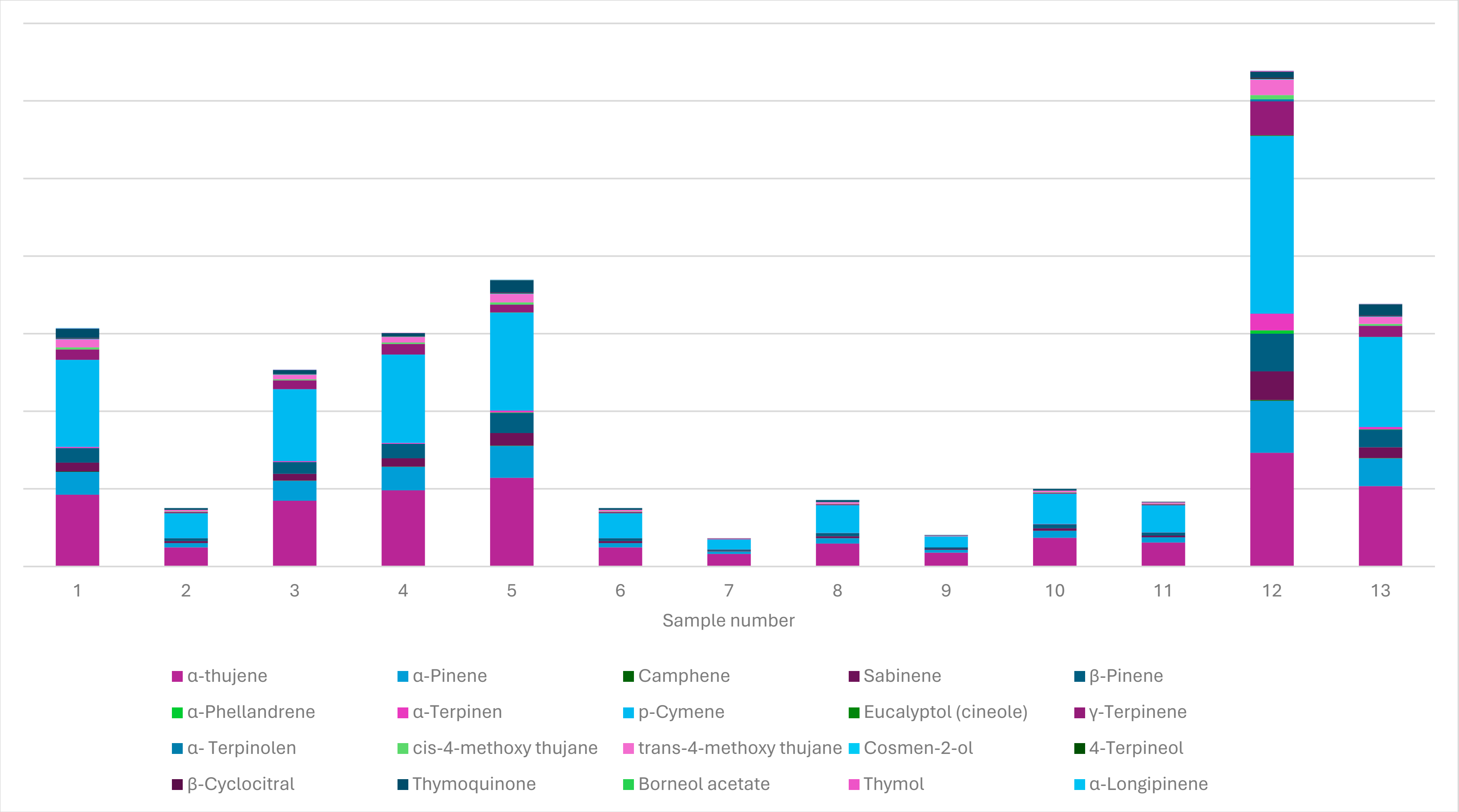

Supplement: Supplementary file 1 [file molecules-29-01360-s001.zip › molecules-2887397-supplementary.pdf]
